# Alcohol-related Outcomes and All-cause Mortality in the Health 2000 Survey by Participation Status and Compared with the Finnish Population

**DOI:** 10.1097/EDE.0000000000001200

**Published:** 2020-04-27

**Authors:** Megan A. McMinn, Linsay Gray, Tommi Härkänen, Hanna Tolonen, Joonas Pitkänen, Oarabile R. Molaodi, Alastair H. Leyland, Pekka Martikainen

**Affiliations:** From the a MRC/CSO Social and Public Health Sciences Unit, University of Glasgow, Glasgow, United Kingdom; b Finnish Institute for Health and Welfare (THL), Helsinki, Finland; c Population Research Unit, Faculty of Social Sciences, University of Helsinki, Finland

**Keywords:** Alcohol consumption, Bias, Finland, Health 2000 Survey, Non-participation, Population sample

## Abstract

In the context of declining levels of participation, understanding differences between participants and non-participants in health surveys is increasingly important for reliable measurement of health-related behaviors and their social differentials. This study compared participants and non-participants of the Finnish Health 2000 survey, and participants and a representative sample of the target population, in terms of alcohol-related harms (hospitalizations and deaths) and all-cause mortality.

**Methods:**

We individually linked 6,127 survey participants and 1,040 non-participants, aged 30–79, and a register-based population sample (n = 496,079) to 12 years of subsequent administrative hospital discharge and mortality data. We estimated age-standardized rates and rate ratios for each outcome for non-participants and the population sample relative to participants with and without sampling weights by sex and educational attainment.

**Results:**

Harms and mortality were higher in non-participants, relative to participants for both men (rate ratios = 1.5 [95% confidence interval = 1.2, 1.9] for harms; 1.6 [1.3, 2.0] for mortality) and women (2.7 [1.6, 4.4] harms; 1.7 [1.4, 2.0] mortality). Non-participation bias in harms estimates in women increased with education and in all-cause mortality overall. Age-adjusted comparisons between the population sample and sampling weighted participants were inconclusive for differences by sex; however, there were some large differences by educational attainment level.

**Conclusions:**

Rates of harms and mortality in non-participants exceed those in participants. Weighted participants’ rates reflected those in the population well by age and sex, but insufficiently by educational attainment. Despite relatively high participation levels (85%), social differentiating factors and levels of harm and mortality were underestimated in the participants.

## Abstract 2

Supplemental Digital Content is available in the text.

The prevalence of various health-related behaviors within a population may be estimated using population-sampled health surveys. Such estimates are critical for formulating and evaluating policies aimed at improving and maintaining population health and wellbeing. Health surveys are typically sampled with the aim of being as representative of the target population as possible, to enable the accurate monitoring of trends in health-related behaviors and the assessment of policy impacts at the population level.^1^ However, declines in levels of participation may threaten the external validity of obtained estimates which are purported to be representative.^2,3^ Survey weights have been used previously to compensate for any bias introduced through non-participation;^4,5^ however, there is mixed evidence as to how accurately they are able to reflect the target population and yield estimates within socioeconomic groups.^6–9^ Where participants and non-participants are alike in terms of the weighting characteristics but not the outcome measure of interest (which, in general terms, could be a mean or proportion of a variable, or association between characteristics), sufficient correction for non-participation bias cannot be gained through weighting methods.^10^ However, typically little is known about those who do not participate. Direct comparisons between participants and non-participants are only possible in a limited number of settings, such as Nordic countries, which have unique population identifiers and potential for comprehensive record linkage. Where non-participants can be identified, they typically differ from participants on baseline sociodemographic characteristics.^11^ However, there is mixed evidence as to whether there are differences in health behaviors or status between participants and non-participants, with some finding no distortion,^12,13^ whilst others find substantial differences overall and within subgroups.^8,14,15^ Such non-participation biases may lead to underestimates in the population prevalence of health behaviors,^8^ and the biases may be particularly severe for alcohol consumption and alcohol-related harm.^16^ We therefore chose alcohol-related harm as the exemplar topic in these analyses.

Amongst other health behaviors, alcohol consumption may influence the decision to participate in a health survey. Hazardous and harmful drinkers may be difficult to recruit,^7^ and their non-participation may bias downward the estimates of alcohol-consumption for the whole population. Differences in alcohol-related morbidity and mortality between participants and non-participants have previously been investigated in Finland,^15,17^ and Denmark^8^ with notably higher rates of harms for non-participants, suggesting that they may consume more alcohol than participants in general.^18^ Comparisons of participants and the general population have revealed substantially lower rates of all-cause mortality and alcohol-related harms in participants,^7,9^ indicating that the participating survey sample is systematically non-representative of the target population. Rates of alcohol-related mortality have been found to be larger in manual, compared to non-manual occupations, over a number of years.^19^ At the same time, socioeconomic differences in self-reported alcohol consumption among survey participants have often been modest,^20^ raising the possibility that survey-based estimates of consumption differentials are at least partly related to non-participation biases.^21^

This article aims to provide the first combined comparison of alcohol-related harms and all-cause mortality in survey participants to (1) non-participants, and (2) to the target population. Comparison (1) enables an assessment of the extent of non-participation bias in terms of health outcomes, and adds to the small evidence base of studies able to make such comparisons. Comparison (2) explores whether the post-survey weights are sufficient to reconcile any differences between the health survey participants alone and the target population (i.e., does any non-participation bias found in (1) affect the external validity of the survey). Previous research on non-participation bias is typically based upon samples with low levels of participation; however, a high level of participation does not necessarily equate to a study that is generalizable of the target population by socioeconomic status, and free of non-participation bias.^22^ As the health survey under examination has a relatively high level of participation, it will be of further interest to assess whether there is evidence of a non-participation bias.

## METHODS

### Data Sources

#### Health 2000

The Health 2000 survey was conducted in Finland, with interviews and health examinations taking place between August 2000 and June 2001.^23^ The sample was constructed to be nationally representative and was drawn using two-stage cluster sampling to identify those aged 30 and over, with an oversample of those aged over 80. The selected sample (“survey sample”; n = 8,028) were invited to participate in an interview conducted in their place of residence and attend a health examination at a local health center. The survey sample achieved a high overall participation rate; 92.9% of the study sample aged 30+ participated in at least one data collection component of the study. Given that multiple co-morbidities are more common at the older ages, and patterns of alcohol consumption are more likely to change with age,^24^ the sample used in this analysis was restricted to those aged 30–79 years at baseline, resulting in a lowered participation level of 85%.

Alcohol consumption information was collected through a self-completed questionnaire given to participants after the home-interview. Participants for this analysis were identified as those who had completed the questionnaire, and non-participants were those who had either not returned it, but had participated in other parts of the data collection, or had not participated in any part of the survey. The start of follow-up for participants was the date of the home-interview (August 2000 to June 2001), while the date of invitation to participate was used for non-participants.

Sampling weights to correct for non-participation were available based on age group, sex, stratum and cluster allocations, and spoken language of participants. Additionally, age at baseline and sex were available from the sampling frame for the survey sample.

#### General Population Data

Statistics Finland constructed an 11% random sample of the population aged 15 or more, permanently residing in Finland at the end of any of the years 1987–2007 (N = 602,151). We limited our analyses to those 30–79-year-old alive on 20 October 2000 (median baseline date for Health 2000 cohort, N = 496,079).

#### Measurement of Education

Educational attainment, defined as level of education completed, for the survey and population samples was extracted from a population register maintained by Statistics Finland and coded using the Finnish Standard Classification 1997^25^ for the survey sample and the equivalent 2007 classification^26^ for the population sample. Both the population and survey samples education pertains to status on December 31, 2000, except for those in the population sample who died during 2000; they were assigned their attainment for the year prior as a proxy. Educational attainment was collapsed into three categories: primary, secondary (e.g., high school), and tertiary (e.g., bachelor level degree).

#### Linked Morbidity and Mortality Records

The outcomes of interest in this analysis are rates of alcohol-related harms (hospitalizations and deaths due to alcohol-related causes) and all-cause mortality. Records of alcohol-related inpatient hospitalizations and all deaths were individually linked to the Health 2000 survey data. Records of hospitalizations were extracted from the Care Register for Health Care, controlled by the National Institute for Health and Welfare (THL, Finnish: Terveyden ja hyvinvoinnin laitos). The register contains records of hospital discharges from 1969 onwards; however, our extract was restricted to instances from 1996 to 2012, coinciding with the introduction of International Classification of Diseases version 10 in Finland. The database includes the main diagnosis, and additional symptoms as reasons for hospitalization, with dates of admission and discharge. We excluded outpatient specialist hospitalizations from the analyses.

Records of all deaths occurring in the survey sample until the end of 2012 were obtained from Statistics Finland. Between being identified for inclusion into the survey, and the start of fieldwork, 49 persons (ages 30+ years) had died and were excluded from all analyses. Two individuals (one of each participation status) were known to have died; however, the cause of death was not available, as their deaths had occurred outside Finland. As the dates of death were available, these individuals were included in the counts of all-cause mortality.

For the general population sample, we obtained linkage to hospitalization and death records registered until the end of 2012 from the same sources, and Statistics Finland made aggregate counts available.

Survey participants had provided informed consent for record linkage. For non-participants and the population sample, consent was not required as the register data were used for statistical and scientific purposes.^27^ Therefore, hospitalization and mortality records for all participants, non-participants, and the population sample are available for analysis. The codes used to define alcohol-related harms are available in eTable 1; http://links.lww.com/EDE/B667, and comprise conditions that are fully attributable to alcohol use, or for which harmful alcohol consumption is a contributing factor.^28^

#### Ethics

The Ethical Committee for Research in Epidemiology and Public Health at the Hospital District of Helsinki and Uusimaa Ethics provided approval for the Health 2000 survey (No. 407/E3/2000). All survey participants provided written informed consent. Access to the Health 2000 survey dataset, and linked hospitalization and death records were granted by the Statistics Finland and National Institute for Health and Welfare (THL) in Finland.

### Analytic Strategy

Differences in incident alcohol-related harms and all-cause deaths between survey participants and non-participants, and survey participants and the population sample were quantified using Poisson regression. Fractional years to first alcohol-related event after baseline was used as the offset in the estimation of rates of alcohol-related harms, whereas fractional years to death was used in the all-cause mortality estimates. Age-standardized rates of alcohol-related harms and all-cause mortality were based on the European Standard Population 1976,^29^ given its closer reflection of the Finnish population structure within the ages of 30 and 79 years at the year 2000, compared to the 2013 European Standard Population. In addition, the use of this standard population offers the opportunity for wider comparisons than by standardizing to the Finnish population alone. In calculating the incidence rates of alcohol-related harms, we confined the numerator and denominator data to those with no previous alcohol-related hospitalizations occurring between January 1, 1996 and baseline. Those with previous alcohol-related hospitalizations were included in the analyses of all-cause mortality. The numerator and denominator data were aggregated by sex, 5-year age group, level of educational attainment and source (survey sample participants, non-participants, and population sample). Analyses were performed incorporating the sampling weights for participants and adjusted to allow for robust standard errors.

Rates and rate ratios (RR) of harms and mortality were estimated using a 5-year age group and an indicator for the source in a Poisson regression model, and were tested for goodness of fit.^30^ We reanalyzed all Poisson models using negative binomial regression, and found them to be consistent with the original estimates. RR within levels of educational attainment were estimated by the inclusion of an interaction between the education level and source, and those estimated in the comparisons with the population sample were predicted using negative binomial regression, due to over-dispersion in the data.^31^ We repeated all analyses by sex and performed in Stata 14.1 (StataCorp, College Station, TX).^32^ Syntax is available as an eAppendix; http://links.lww.com/EDE/B667.

### Sensitivity Analyses

We performed two extensions to the analyses. First, we estimated rates and RR for any alcohol-related harm, for both comparisons. ‘Any alcohol-related harm’ comprises any alcohol-related hospitalization or death occurring between baseline and the end of 2012, and differs from the incident harms presented in the main analyses as those who had been previously hospitalized between January 1, 1996 and baseline are now included if they had experienced an event during follow-up (see eTables 2–4; http://links.lww.com/EDE/B667). Second, we repeated all analyses excluding the weights in order to explore the effects of weighting for non-participation on the results (see eTables 5–8; http://links.lww.com/EDE/B667).

## RESULTS

Of the 8,028 individuals selected into the Health 2000 sample, 7,191 were aged between 30 and 79 years at baseline. Following selection, 24 individuals died prior to interview and we removed them from further analysis. Of the remaining the sample, 6,127 (85%) participated by completing the health questionnaire, and 1,040 (15%) were categorized as non-participants. While the sample as a whole contained more women (n = 3,758, 52%), a larger proportion of the non-participants were male (n = 569, 55%). There was a greater percentage of both younger (30–34 years: 13% vs. 11%) and older (75–79 years: 7% vs. 5%) non-participants, compared to participants. A greater percentage of participants had attained secondary (35% vs. 32%) and tertiary (29% vs. 23%) levels of education, while a lower percentage of participants had a primary level of education (37% vs. 46%).

Table 1 presents the numbers of alcohol-related harms and all-cause deaths for participants and non-participants, by educational attainment and sex. Crude rates of all outcomes in non-participants exceeded those of participants in all combinations of sex and attainment, except for men with secondary (all-cause mortality) and tertiary education (incident harms).

**TABLE 1.**
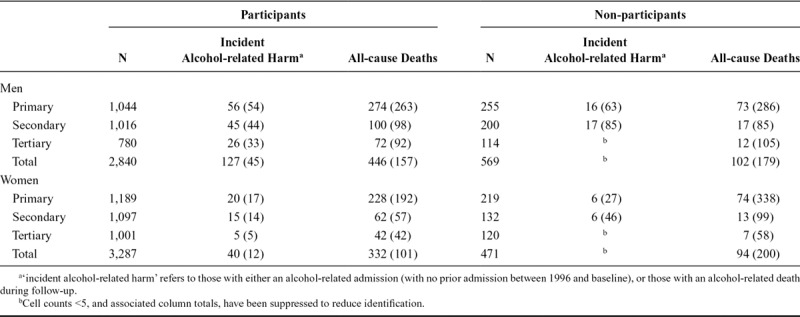
Numbers (Crude Rate Per 1,000) of Incident Alcohol-Related Harms and All-Cause Mortality by Participation Status, Sex and Educational Attainment, Men and Women Aged 30–79 Years at Baseline

Table 2 reveals that rates of incident alcohol-related harms were, for men, 1.5 (95% confidence interval [CI] = 1.2, 1.9), and for women, 2.7 (95% CI = 1.6, 4.4) times larger among non-participants, compared to participants. The ratios for all-cause mortality revealed similar associations for men and women (RR = 1.6 for men and 1.7 for women), with rates within non-participants again exceeding those of participants.

**TABLE 2.**
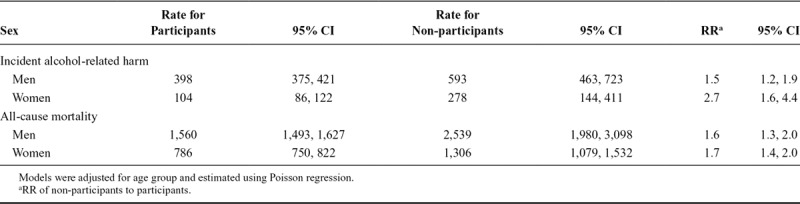
Age-Standardized Rates and RR of Alcohol-Related Harm and All-Cause Mortality Per 100,000 Person Years at Risk Among Weighted Participants and Non-Participants of the Health 2000 Survey Aged 30–79 Years

The rates and RR comparing outcomes among non-participants relative to participants within each level of educational attainment are given in Table 3. Rates of harms declined progressively with increasing levels of educational attainment in the participants, while in non-participants rates of harms for those with secondary levels of education exceeded rates for those with primary and tertiary levels. In terms of RR, the only level of education to exhibit large differences between non-participants and participants in the incident alcohol-related harms is primary (women only RR = 1.7, 95% CI = 1.0, 3.0) and secondary (men RR = 2.1, 95% CI = 1.4, 3.1; women RR = 3.4, 95% CI = 1.2, 9.9) levels of education. Rates of all-cause mortality in non-participants exceed those of participants in all levels of educational attainment.

**TABLE 3.**
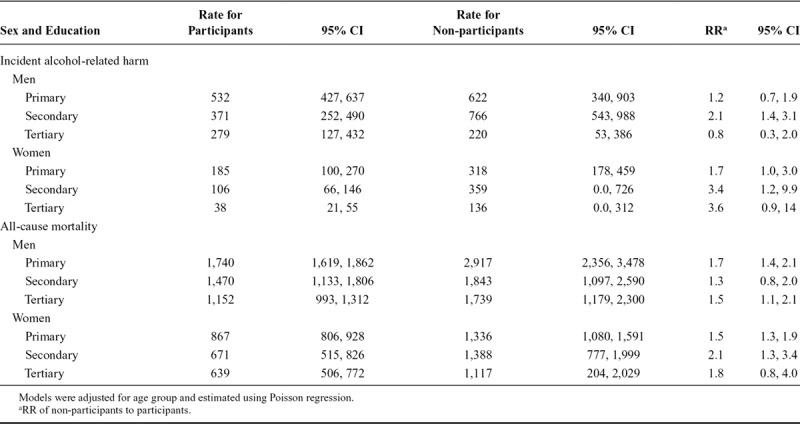
Age-Standardized Rates and RR of Alcohol-Related Harms and All-Cause Mortality for Non-Participants Compared to Weighted Participants by Educational Attainment

Comparisons of the Finnish population and survey participants are reported in Table 4. Rates of incident alcohol-related harms were slightly higher in male participants compared to the population sample (RR = 1.0).Rates of all-cause mortality were consistently higher in the population sample for both sexes (all RR > 1).

**TABLE 4.**
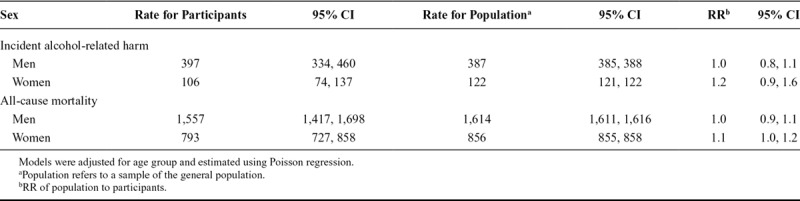
Age-Standardized Rates and RR of Alcohol-Related Harm and All-Cause Mortality Per 100,000 Person Years at Risk Among Weighted Participants of the Health 2000 Survey and the Finnish Population Aged 30–79 Years

Comparisons of educational differentials between participants and the population sample, described in Table 5, were mixed. RR revealed women in the population with tertiary levels of education had rates of incident alcohol related harms 1.8 (95% CI = 1.1, 3.2) times those in the participant sample, whilst men in the same educational category were 0.8 (95% CI = 0.4, 1.4) times lower than the participant sample.

**TABLE 5.**
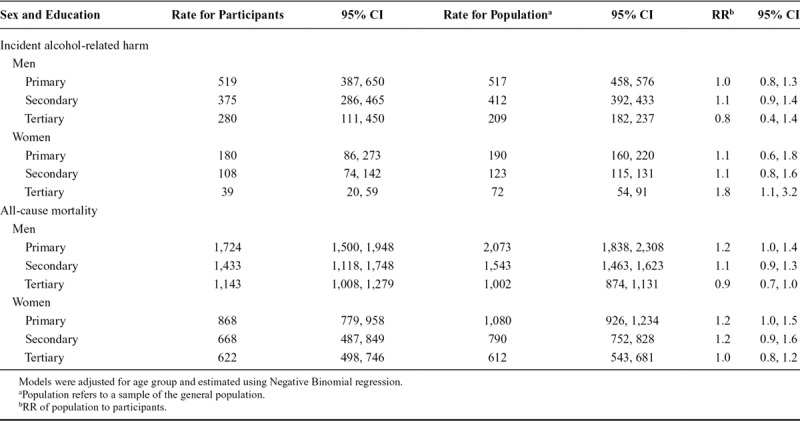
Age-Standardized Rates and RR of Alcohol-Related Harms and All-Cause Mortality for the Finnish Population Compared to Weighted Participants by Educational Attainment

All-cause mortality rates in the population exceeded those of the participants at primary and secondary levels of education, but not tertiary. The RR declined with increasing levels of education for both sexes.

Estimated rates and rate-ratios of any alcohol-related harm were generally higher than those estimated for the incident harms (see eTables 2–4; http://links.lww.com/EDE/B667). Analyses were repeated ignoring sampling weights for participants, in order to investigate the effectiveness of post-hoc weighting strategies (see eTables 5–8; http://links.lww.com/EDE/B667). Given the relatively high participation levels (85%), it is not surprising that there was little difference in the point estimates; however, the width of the CIs increased, due to the allowance of robust standard errors in the weighted models.

## DISCUSSION

To our knowledge, this is the first study to present comparisons between participants and non-participants, and participants and a population sample together. As the direct comparison of survey participants and non-participants is only possible in a limited number of settings, this study represents a rare quantification of differences in terms of mortality and alcohol-related harms. Only three previous studies that we know of have investigated differences in alcohol-related morbidity between participants and known non-participants of health surveys.^8,15,17^ They found, in agreement with our study, that non-participants experience higher rates of alcohol-related harms and all-cause mortality, compared to participants. A further novelty of our analysis is that it illustrates the presence of non-participation bias in a health survey with very high levels of participation.

For participants and the population sample, the rates of alcohol-related harms decreased with increasing educational attainment, for both men and women. In the non-participants, rates of harms in those with secondary education exceeded those with primary levels of education, indicating a potentially different relationship between alcohol consumption and education in the non-participants, compared to the participants and the general population.

The RR of incident alcohol-related harms, and all-cause mortality for men and women, indicate that rates were higher in non-participants compared to participants. RR within educational attainment generally indicated higher rates of harms and deaths in the non-participants, compared to the participants across the gradient; however, there were some inconclusive results. The sensitivity analyses of alcohol-related harms revealed that persons who had experienced an alcohol-related hospitalization prior to survey recruitment were more likely to be non-participants, based on the increased RR.

Comparisons between survey participants and a sample of the general population revealed that rates of any alcohol-related harms and all-cause mortality were higher in the population, compared to participants, although the majority of findings within levels of educational attainment were inconclusive as to whether or not a difference was present. This may reflect the high response rate of 85% in this survey sample. However, the instances where large differences between participants and the population sample were found (women with tertiary levels of education in the case of harms, and men and women with primary levels of education in all-cause mortality), point to an insufficiency in the use of weighting to adjust for non-participation, which is likely to hold in settings other than Finland. The RR of incident alcohol-related harms for males in the population to the participants was estimated to be less than 1, a surprising finding given previous research.^18^ Rates of all-cause mortality in the population sample were between 20% and 30% greater than the survey participants with primary levels of attainment, but this difference decreased with increasing levels of educational attainment. This points to systematic differences between participants and the population among those with low education. Comparisons of health behaviors or outcomes by participation status, and between participants and total populations, have previously been investigated in other studies with lower levels of participation.^8,12,15,33,34^ In Finland, hazard ratios of all-cause mortality were found to be higher in non-participants, both overall and within measures of occupational class and education.^12^ Similarly in Denmark, non-participants had increased hazard ratios of both alcohol-related mortality and morbidity.^8^

The strengths of this study include the use of nationally representative data, with 12 years of complete, individually linked follow-up data available for participants, non-participants, and a population sample. The comparisons between participants and the population sample are a further strength, with the sex-specific and within levels of education comparisons revealing that weighting for non-participation was not sufficient across the socioeconomic spectrum.

There are also several limitations to consider. First, although sampled individuals with prior alcohol-related hospitalizations were removed from the analysis of incident alcohol-related harms, details of previous hospitalizations were only available from 1996 onwards. Given that there is some evidence that individuals previously hospitalized for alcohol-related conditions are more likely to become non-responders to health surveys,^35^ the rates of incident alcohol-related harms within this survey sample, and for the non-participants, in particular, may be slightly over-estimated if there were any additional hospitalizations prior to 1996. Second, due to the small numbers of alcohol-related harms that had occurred during the follow-up period, this study may not have had sufficient power to detect differences, especially within some of the educational attainment groups. Finally, no adjustments were made to take the hierarchical nature of the Health 2000 sample into account, given that the cohort was cluster sampled from 80 health center districts. Therefore, there may be unmeasured intraclass correlations.

Future research using a different health survey, or a series of health surveys, with a larger sample size, may be able to provide an assessment of the differences in the time trends in rates between participants and non-participants. Due to the small numbers of events in some years, especially within levels of educational attainment, we were unable to include this.

This study highlights the importance of representative sampling and illustrates the potential effects of non-participation bias, even with a high level of participation, given the large differences in rates of alcohol-related harms and all-cause mortality between participants and non-participants. In conclusion, rates of alcohol-related harms and all-cause mortality in non-participants were found to exceed those of participants, whilst participants’ rates reflected those in the population well in terms of age and sex, but insufficiently within educational attainment. This study demonstrates that despite relatively high levels of participation, non-participation can bias results, particularly in those with lower levels of education. These findings have implications for the use of health surveys to estimate the prevalence of health behaviors across the socioeconomic gradient, given the non-participation biases found in analyzing health outcomes.

## ACKNOWLEDGMENTS

We would like to thank the participants of the Health 2000 study, National Institute for Health and Welfare (THL) and Statistics Finland for the provision of the sociodemographic, hospitalization, and death data. Thanks in particular to Harri Rissanen from the National Institute for Health and Welfare (THL) for the preparation and provision of the linked Health 2000 survey data.

## Supplementary Material


