# Supplementary material for: Alcohol-related Outcomes and All-cause Mortality in the Health 2000 Survey by Participation Status and Compared with the Finnish Population
Source: Epidemiology. 2020 Apr 27;31(4):534–41. doi: 10.1097/EDE.0000000000001200 (PMC7269017; doi:10.1097/EDE.0000000000001200)
Supplement: Supplementary file 1 [file ede-31-534-s001.docx]

| Diseases and Related Health Problems | ICD 10 |
| --- | --- |
| Alcohol induced Pseudo-Cushing’s syndrome | E24.4 |
| Wernicke’s Encephalopathy | E51.2 |
| Mental and behavioural disorders due to use of alcohol | F10.0 – F10.9 |
| Degeneration of nervous system due to alcohol | G31.2 |
| Special epileptic syndromes - related to alcohol | G40.51 |
| Alcoholic polyneuropathy | G62.1 |
| Alcoholic myopathy | G72.1 |
| Alcoholic cardiomyopathy | I42.6 |
| Alcoholic gastritis | K29.2 |
| Alcohol liver diseases | K70.0 – K70.9 |
| Chronic hepatitis, not elsewhere classified | K73.0 – K73.9 |
| Hepatic fibrosis | K74.0 |
| Hepatic sclerosis | K74.1 |
| Hepatic fibrosis with hepatic sclerosis | K74.2 |
| Other and unspecified cirrhosis of liver | K74.6 |
| Alcohol-induced chronic pancreatitis | K86.0 |
| Maternal care for (suspected) damage to fetus from alcohol | O35.4 |
| Fetus and newborn affected by maternal use of alcohol | P04.3 |
| Fetal alcohol syndrome (dysmorphic) | Q86.0 |
| Finding of alcohol in blood | R78.0 |
| Toxic effect of ethanol | T51.0 |
| Toxic effect of methanol | T51.1 |
| Toxic effect of alcohol; unspecified | T51.9 |
| Accidental poisoning by and exposure to alcohol | X45.0 – X45.9 |
| Intentional self-poisoning by and exposure to alcohol | X65.0 – X65.9 |
| Poisoning by and exposure to alcohol, undetermined intent | Y15.0 – Y15.9 |
| Alcohol deterrents | Y57.3 |
| Evidence of alcohol involvement determined by blood alcohol level | Y90 |
| Evidence of alcohol involvement determined by level intoxication | Y91 |
| Alcohol rehabilitation | Z50.2 |
| Alcohol abuse counselling and surveillance | Z71.4 |
| Alcohol Use | Z72.1 |

eTable 1: Alcohol-related diseases and health problems with ICD 10 codes

eTable 2: Numbers (crude rate per 1,000) of any alcohol related harms by participation status, sex and educational attainment, men and women aged 30 – 79 years at baseline.

|  | **N** | **Participants** | **N** | **Non-participants** |
| --- | --- | --- | --- | --- |
| **Men** | 2,840 | 159 (56.0) | 569 | ^a^ |
| Primary | 1,044 | 72 (69.0) | 255 | 20 (78.4) |
| Secondary | 1,016 | 60 (59.1) | 200 | 22 (110.0) |
| Tertiary | 780 | 27 (34.6) | 114 | ^a^ |
|  |  |  |  |  |
| **Women** | 3,287 | 47 (14.3) | 471 | ^a^ |
| Primary | 1,189 | 21 (17.7) | 219 | 7 (32.0) |
| Secondary | 1,097 | 20 (18.2) | 132 | 8 (60.6) |
| Tertiary | 1,001 | 6 (6.0) | 120 | ^a^ |
| ^b^ Cell counts < 5, and associated column totals, have been suppressed to reduce identification | | | | |

eTable 3: Age-standardised rates and rate ratios of any alcohol-related harm per 100,000 person years at risk among weighted participants and non-participants of the Health 2000 Survey aged 30 - 79 years. Models were adjusted for age group and estimated using Poisson regression.

| Sex | Rate for participants | | 95%CI | | | Rate for non-participants | | 95%CI | | Rate Ratio ^a^ | 95%CI |
| --- | --- | --- | --- | --- | --- | --- | --- | --- | --- | --- | --- |
| Men | | 495.8 | | 470.4, 521.2 | 750.2 | | 604.6, 895.8 | | 1.51 | | 1.23, 1.86 |
| Primary | | 686.4 | | 582.0, 790.8 | 769.6 | | 522.4, 1016.9 | | 1.12 | | 0.8, 1.6 |
| Secondary | | 490.1 | | 370.9, 609.3 | 971.9 | | 778.4, 1165.4 | | 1.98 | | 1.5, 2.7 |
| Tertiary | | 288.1 | | 126.5, 449.8 | 293.0 | | 135.5, 450.6 | | 1.02 | | 0.5, 2.2 |
| Women | | 122.0 | | 103.3, 140.8 | 359.5 | | 217.9, 501.1 | | 2.95 | | 1.94, 4.48 |
| Primary | | 190.0 | | 101.8, 278.2 | 371.8 | | 207.8, 535.9 | | 1.96 | | 1.1, 3.4 |
| Secondary | | 140.4 | | 88.6, 192.1 | 483.1 | | 153.8, 812.4 | | 3.44 | | 1.6, 7.3 |
| Tertiary | | 46.3 | | 28.0, 64.5 | 203.5 | | 25.5, 381.4 | | 4.4 | | 1.7, 11.2 |

| ^a^ Rate Ratio of non-participants to participants |
| --- |

|  |  |  |  |  |  |  |
| --- | --- | --- | --- | --- | --- | --- |

eTable 4: Age-standardised rates and rate ratios of alcohol-related harm per 100,000 person years at risk among weighted participants of the Health 2000 Survey and the Finnish population aged 30 - 79 years. Models were adjusted for age group and estimated using Poisson regression.

| Sex | Rate for participants | | 95%CI | | | Rate for Population | | 95%CI | | Rate Ratio ^a^ | 95%CI |
| --- | --- | --- | --- | --- | --- | --- | --- | --- | --- | --- | --- |
| Men | | 494.3 | | 416.2, 572.3 | 502.7 | | 501.2, 504.3 | | 1.02 | | 0.87, 1.19 |
| Primary | | 665.8 | | 490.6, 841.1 | 680.4 | | 588.5, 772.4 | | 1.02 | | 0.8, 1.4 |
| Secondary | | 494.8 | | 394.7, 594.8 | 537.0 | | 501.3, 572.7 | | 1.09 | | 0.9, 1.3 |
| Tertiary | | 288.9 | | 122.2, 455.5 | 268.3 | | 223.6, 312.9 | | 0.93 | | 0.5, 1.7 |
| Women | | 123.7 | | 92.2, 155.1 | 152.9 | | 152.2, 153.5 | | 1.24 | | 0.96, 1.59 |
| Primary | | 190.5 | | 97.9, 283.0 | 247.6 | | 206.7, 288.5 | | 1.30 | | 0.8, 2.2 |
| Secondary | | 142.6 | | 101.7, 183.6 | 147.4 | | 138.0, 156.7 | | 1.03 | | 0.8, 1.4 |
| Tertiary | | 47.7 | | 21.8, 73.6 | 95.4 | | 68.4, 122.4 | | 2.00 | | 1.1, 3.7 |

| ^a^ Rate Ratio of population to participants |
| --- |

eTable 5: Age-standardised rates and rate ratios of alcohol-related harm and all-cause mortality per 100,000 person years at risk among participants and non-participants of the Health 2000 Survey aged 30 - 79 years. No survey weights were incorporated in the model.

| Sex | Rate for participants | 95%CI | Rate for non-participants | 95%CI | Rate Ratio ^a^ | 95%CI |
| --- | --- | --- | --- | --- | --- | --- |
| *Incident alcohol-related harm* | | | | | | |
| Men | 397.8 | 328.1, 467.5 | 593.2 | 397.4, 788.9 | 1.49 | 1.03, 2.16 |
| Women | 104.3 | 71.9, 136.7 | 277.4 | 129.8, 424.0 | 2.66 | 1.44, 4.92 |
| *All alcohol-related harm* | | | | | | |
| Men | 495.6 | 418.0, 573.2 | 750.7 | 531.6, 969.7 | 1.51 | 1.09, 2.11 |
| Women | 122.6 | 87.5, 157.7 | 359.3 | 190.7, 527.8 | 2.93 | 1.69, 5.07 |
| *All-cause mortality* | | | | | | |
| Men | 1560.3 | 1413.1, 1707.5 | 2545.7 | 2041.0, 3050.3 | 1.63 | 1.31, 2.03 |
| Women | 785.9 | 700.8, 870.9 | 1310.6 | 1038.5, 1582.8 | 1.67 | 1.32, 2.10 |
| ^a^ Rate Ratio of non-participants to participants. | | | | | | |

eTable 6: Rate Ratios of alcohol-related harms and all-cause mortality for non-participants compared to participants by educational attainment. No survey weights were incorporated in the model.

| **Sex and Education** | **Rate for participants** | **95%CI** | **Rate for non-participants** | **95%CI** | **Rate Ratio** ^a^ | **95%CI** |
| --- | --- | --- | --- | --- | --- | --- |
| *Incident alcohol-related harms* | | | | | | |
| **Men** | | | | | | |
| Basic | 535.9 | 390.2, 681.6 | 622.1 | 316.7, 927.6 | 1.16 | 0.7, 2.0 |
| Secondary | 371.2 | 260.9, 481.5 | 765.2 | 394.4, 1136.1 | 2.06 | 1.2, 3.6 |
| Tertiary | 274.4 | 168.2, 380.7 | 219.9 | 0.0, 469.1 | 0.80 | 0.2, 2.7 |
| **Women** |  |  |  |  |  |  |
| Basic | 183.7 | 94.4, 273.0 | 317.8 | 54.4, 581.2 | 1.73 | 0.7, 4.3 |
| Secondary | 108.6 | 53.4, 163.9 | 357.8 | 66.7, 648.9 | 3.29 | 1.3, 8.6 |
| Tertiary | 37.6 | 4.5, 70.7 | 135.5 | 0.0, 324.3 | 3.61 | 0.7, 18.6 |
| *Any alcohol-related harms* | | | | | | |
| **Men** | | | | | | |
| Basic | 691.6 | 525.5, 857.8 | 770.2 | 432.1, 1108.4 | 1.11 | 0.7, 1.8 |
| Secondary | 488.7 | 362.9, 614.4 | 971.1 | 557.5, 1384.7 | 1.99 | 1.2, 3.3 |
| Tertiary | 283.8 | 176.1, 391.4 | 293.5 | 5.4, 581.6 | 1.03 | 0.4, 3.0 |
| **Women** |  |  |  |  |  |  |
| Basic | 189.0 | 101.0, 277.0 | 372.1 | 87.3, 657.1 | 1.97 | 0.8, 4.7 |
| Secondary | 143.9 | 80.5, 207.4 | 481.6 | 141.9, 821.2 | 3.35 | 1.5, 7.7 |
| Tertiary | 45.7 | 8.9, 82.4 | 203.2 | 0.0, 434.8 | 4.45 | 1.1, 17.8 |
| *All-cause mortality* | | | | | | |
| **Men** | | | | | | |
| Basic | 1,736.9 | 1527.4, 1946.5 | 2,920.0 | 2248.0, 3592.0 | 1.68 | 1.3, 2.2 |
| Secondary | 1,476.8 | 1172.8, 1780.7 | 1,853.8 | 948.6, 2759.0 | 1.26 | 0.8, 2.1 |
| Tertiary | 1,153.6 | 882.9, 1424.3 | 1,748.2 | 749.0, 2747.5 | 1.52 | 0.8, 2.8 |
| **Women** |  |  |  |  |  |  |
| Basic | 865.4 | 747.1, 983.6 | 1,342.4 | 1022.1, 1662.6 | 1.55 | 1.2, 2.0 |
| Secondary | 674.2 | 504.2, 844.1 | 1,386.8 | 628.0, 2145.7 | 2.06 | 1.1, 3.8 |
| Tertiary | 640.3 | 442.6, 838.0 | 1,113.6 | 281.5, 1945.6 | 1.74 | 0.8, 3.8 |
| ^a^ Rate Ratio of non-participants to participants | | | | | | |

eTable 7: Age-standardised rates and rate ratios of alcohol-related harm and all-cause mortality per 100,000 person years at risk among participants of the Health 2000 Survey and the Finnish population aged 30 - 79 years. No survey weights for the participants were incorporated in the model.

| Sex | Rate for participants | 95%CI | Rate for population ^a^ | 95%CI | Rate Ratio ^b^ | 95%CI |
| --- | --- | --- | --- | --- | --- | --- |
| *Incident alcohol-related harm* | | | | | | |
| Men | 396.7 | 327.7, 465.7 | 386.5 | 377.5 395.6 | 0.97 | 0.82, 1.16 |
| Women | 105.5 | 72.8, 138.2 | 121.5 | 116.6, 126.3 | 1.15 | 0.84, 1.57 |
| *Any alcohol-related harm* | | | | | | |
| Men | 494.4 | 417.6, 571.3 | 502.7 | 492.5, 513.0 | 1.02 | 0.87, 1.19 |
| Women | 124.0 | 88.6, 159.5 | 152.9 | 147.4, 158.3 | 1.23 | 0.92, 1.64 |
| *All-cause mortality* | | | | | | |
| Men | 1552.2 | 1408.1, 1696.3 | 1613.8 | 1594.3, 1633.3 | 1.04 | 0.95, 1.14 |
| Women | 789.6 | 704.6, 874.5 | 856.2 | 844.2, 868.3 | 1.08 | 0.97, 1.21 |
| ^a^ Population refers to a sample of the general population.  ^b^ Rate Ratio of population to participants | | | | | | |

eTable 8: Rate Ratios of alcohol-related harms and all-cause mortality for the Finnish population compared to participants by educational attainment. No survey weights for the participants were incorporated in the model.

| **Sex and Education** | **Rate for participants** | **95%CI** | **Rate for population** ^a^ | **95%CI** | **Rate Ratio** ^b^ | **95%CI** |
| --- | --- | --- | --- | --- | --- | --- |
| *Incident alcohol-related harms* | | | | | | |
| **Men** | | | | | | |
| Basic | 522.8 | 390.5, 655.1 | 516.8 | 457.7, 575.9 | 0.99 | 0.8, 1.3 |
| Secondary | 375.0 | 282.8, 467.2 | 412.2 | 392.0, 432.5 | 1.10 | 0.9, 1.4 |
| Tertiary | 275.7 | 110.6, 440.8 | 209.2 | 181.8, 236.7 | 0.76 | 0.4, 1.4 |
| **Women** |  |  |  |  |  |  |
| Basic | 177.5 | 83.5, 271.6 | 189.9 | 159.9, 219.9 | 1.07 | 0.6, 1.8 |
| Secondary | 110.5 | 75.8, 145.2 | 123.1 | 115.0, 131.3 | 1.11 | 0.8, 1.5 |
| Tertiary | 39.3 | 19.3, 59.4 | 72.2 | 53.9, 90.6 | 1.84 | 1.0, 3.3 |
| *Any alcohol-related harms* | | | | | | |
| **Men** | | | | | | |
| Basic | 671.1 | 494.3, 847.9 | 680.1 | 588.7, 771.4 | 1.01 | 0.8, 1.4 |
| Secondary | 493.8 | 392.3, 595.3 | 536.7 | 501.6, 571.8 | 1.09 | 0.9, 1.4 |
| Tertiary | 285.4 | 122.3, 448.4 | 267.8 | 223.7, 311.8 | 0.94 | 0.5, 1.7 |
| **Women** |  |  |  |  |  |  |
| Basic | 188.8 | 95.6, 281.9 | 247.6 | 206.8, 288.5 | 1.31 | 0.8, 2.2 |
| Secondary | 146.0 | 104.1, 188.0 | 147.4 | 138.0, 156.7 | 1.01 | 0.8, 1.4 |
| Tertiary | 47.1 | 21.2 - 73.0 | 95.4 | 68.4, 122.4 | 2.03 | 1.1,,3.8 |
| *All-cause mortality* | | | | | | |
| **Men** | | | | | | |
| Basic | 1724.1 | 1508.4, 1939.7 | 2072.9 | 1837.9, 2308.0 | 1.20 | 1.0, 1.4 |
| Secondary | 1435.8 | 1131.0, 1740.7 | 1543.3 | 1462.9, 1623.7 | 1.07 | 0.9, 1.3 |
| Tertiary | 1141.4 | 1015.4, 1267.4 | 1003.0 | 875.3, 1130.7 | 0.88 | 0.8, 1.0 |
| **Women** |  |  |  |  |  |  |
| Basic | 866.6 | 779.6, 953.6 | 1079.4 | 925.7, 1233.0 | 1.25 | 1.0, 1.5 |
| Secondary | 669.4 | 496.2, 842.5 | 790.0 | 752.4, 827.6 | 1.18 | 0.9, 1.5 |
| Tertiary | 620.9 | 493.7, 748.1 | 611.6 | 542.6, 680.5 | 0.99 | 0.8, 1.2 |
| ^a^ Population refers to a sample of the general population.  ^b^ Rate Ratio of population to participants | | | | | | |
